# Imaginal Discs – A New Source of Chromosomes for Genome Mapping of the Yellow Fever Mosquito *Aedes aegypti*

**DOI:** 10.1371/journal.pntd.0001335

**Published:** 2011-10-04

**Authors:** Maria V. Sharakhova, Vladimir A. Timoshevskiy, Fan Yang, Sergei Iu. Demin, David W. Severson, Igor V. Sharakhov

**Affiliations:** 1 Department of Entomology, Fralin Life Science Institute, Virginia Tech, Blacksburg, Virginia, United States of America; 2 Institute of Cytology, Russian Academy of Sciences, Saint Petersburg, Russia; 3 Department of Biological Sciences, Eck Institute for Global Health, University of Notre Dame, Notre Dame, Indiana, United States of America; The University of Queensland, Australia

## Abstract

**Background:**

The mosquito *Aedes aegypti* is the primary global vector for dengue and yellow fever viruses. Sequencing of the *Ae. aegypti* genome has stimulated research in vector biology and insect genomics. However, the current genome assembly is highly fragmented with only ∼31% of the genome being assigned to chromosomes. A lack of a reliable source of chromosomes for physical mapping has been a major impediment to improving the genome assembly of *Ae. aegypti*.

**Methodology/Principal Findings:**

In this study we demonstrate the utility of mitotic chromosomes from imaginal discs of 4^th^ instar larva for cytogenetic studies of *Ae. aegypti*. High numbers of mitotic divisions on each slide preparation, large sizes, and reproducible banding patterns of the individual chromosomes simplify cytogenetic procedures. Based on the banding structure of the chromosomes, we have developed idiograms for each of the three *Ae. aegypti* chromosomes and placed 10 BAC clones and a 18S rDNA probe to precise chromosomal positions.

**Conclusion:**

The study identified imaginal discs of 4^th^ instar larva as a superior source of mitotic chromosomes for *Ae. aegypti*. The proposed approach allows precise mapping of DNA probes to the chromosomal positions and can be utilized for obtaining a high-quality genome assembly of the yellow fever mosquito.

## Author Summary

Dengue fever is an emerging health threat to as much as half of the human population around the world. No vaccines or drug treatments are currently available. Thus, disease prevention is largely based on efforts to control its major mosquito vector *Ae. aegypti.* Novel vector control strategies, such as population replacement with pathogen-incompetent transgenic mosquitoes, rely on detailed knowledge of the genome organization for the mosquito. However, the current genome assembly of *Ae. aegypti* is highly fragmented and requires additional physical mapping onto chromosomes. The absence of readable polytene chromosomes makes genome mapping for this mosquito extremely challenging. In this study, we discovered and investigated a new source of chromosomes useful for the cytogenetic analysis in *Ae. aegypti –* mitotic chromosomes from imaginal discs of 4^th^ instar larvae. Using natural banding patterns of these chromosomes, we developed a new band-based approach for physical mapping of DNA probes to the precise chromosomal positions. Further application of this approach for genome mapping will greatly enhance the utility of the existing draft genome sequence assembly for *Ae. aegypti* and thereby facilitate application of advanced genome technologies for investigating and developing novel genetic control strategies for dengue transmission.

## Introduction

*Ae. aegypti* is a principal vector for yellow fever, dengue and chikungunya viruses [1], [2]. These diseases have a significant worldwide impact on human health. Yellow fever affects up to 600 million lives and is responsible for about 30,000 deaths annually [3]. Dengue fever is a threat to >2.5 billion people in tropical and subtropical regions, where between 50 to 100 million infections occur each year [2], [4], [5]. The incidence of dengue fever is increasing globally [6], for example in developed areas like Singapore where dengue was thought to be well-controlled [7] and is a growing threat to the United States [8]. Despite all control campaigns, *Ae. aegypti* has expanded its range to most subtropical and tropical regions during the last several decades. This mosquito prefers to feed on humans and breeds in areas that humans inhabit [9].

To facilitate the development of genome-based strategies for mosquito control, genomes for three major disease vectors--the African malaria mosquito *Anopheles gambiae*, the southern house mosquito *Culex quinquefasciatus,* and the yellow fever mosquito *Ae. aegypti--*have been sequenced [10], [11]. Among genomes of these three species, the genome of *Ae. aegypti* is the largest [11]. The draft genome sequence consists of 1,376 million base pairs, which is ∼5 times larger than the *An. gambiae* genome [10] and ∼2 times larger than the *Cx. quinquefasciatus* genome [12]. About half of the genome consists of transposable elements. The genome shows “short period interspersion” meaning that, in general, ∼1–2 kb fragments of unique sequences alternate with ∼0.2–4 kb fragments of repetitive DNA [13]. Abundance of repetitive elements in the genome leads to low levels of replication and poor spreading of polytene chromosomes of *Ae. aegypti* [14], [15]. The yield of chromosome preparations useful for cytogenetic studies was only 0.5% for salivary glands [15]. At the same time, the large size of the genome makes mitotic chromosomes of this mosquito large and easily identifiable. The average size of the biggest metaphase chromosome in *Ae. aegypti* was estimated as 7.7 µm [16], which is bigger than the average sizes of human metaphase chromosomes and comparable with the size of the human chromosomes at prometaphase [17]. The average size of the biggest human chromosome at prometaphase was estimated as 9.24 µm.

Most of the classical cytogenetic studies on *Ae. aegypti* undertaken in the past were performed on mitotic or meiotic chromosomes from larval brain or male testis [18], [19], [20]. It has been demonstrated that *Ae. aegypti* has a karyotype typical to that found in other mosquitoes and includes three pairs of chromosomes. These chromosomes were originally designated as chromosomes I, II, and III in the order of increasing size [18]. However, later chromosomes were renamed in accordance with *Ae. aegypti* linkage groups as chromosomes 1, 2, and 3 [21]. Chromosome 1 was described as the shortest metacentric chromosome; chromosome 2 as the longest, also a metacentric chromosome; and chromosome 3 as a medium-length submetacentric chromosome with the secondary constriction on the longer arm. However, precise measurement of the centromeric index made on spermatagonial metaphase chromosomes has indicated that all *Ae. aegypti* chromosomes fall into the category of metacentric chromosomes according to the standard classification [22], [23].

Unlike the anophelines, the sex chromosomes are homomorphic in all culicine mosquitoes, including *Ae. aegypti* [18]. The sex determination alleles were linked to chromosome 1 and described as *Mm* in males and *mm* in females [24]. M. Motara and K. Rai proposed to name sex chromosomes as “m” and “M” chromosomes for female and male determining chromosomes, respectively [20]. However, it was also popular to refer to sex chromosomes in *Aedes* as “X” and “Y” [19]. The precise measurement of the sex chromosomes in males and females has indicated that the female chromosome 1 is slightly bigger in size [22]. The C-banding technique has also demonstrated differences between male and female sex chromosomes in *Ae. aegypti* [20]. Typically females have pericentromeric and additional distinct intercalary bands on both chromosomes 1 which are absent on the putative male determining sex chromosome. C-banding has been found to be variable in different strains of *Ae. aegypti*. For example, an intercalary band can be present on the male chromosome in some strains, and intercalary bands may be differ in size in females [25], [26]. The silver staining technique [26] and *in situ* hybridization of 18S and 28S ribosomal genes [27] indicated the location of ribosomal locus on both sex chromosomes of *Ae. aegypti*.

The genetic mapping of the *Ae. aegypti* genome has been conducted in parallel with cytogenetic studies. An early genetic map included about 70 morphological, insecticide-resistance and isozyme markers [28]. Later, additional genetic maps were developed using restriction fragment-length polymorphism (RFLP) markers, random-amplified polymorphic DNA (RAPD) loci, single-strand conformation polymorphism (SSCP), and single-nucleotide polymorphism (SNP) markers [29], [30], [31]. A composite map for RFLP, SSCP, and SNP markers incorporated 146 loci and covered 205 cM [13]. These maps provided the tools to localize a number of quantitative trait loci (QTLs) related to the mosquito's ability to transmit the filarioid nematode *Brugia malayi* [32], the avian malaria parasite *Plasmodium gallinaceum* [33], [34], and dengue virus [35], [36]. Advent of the fluorescent *in situ* hybridization technique allowed mapping of BAC clones, cosmids, and cDNA probes on mitotic chromosomes from the ATC-10 cell line of *Ae. aegypti* [16]. The chromosome positions of these clones were measured by FLpter: a fractional length from the short arm telomeric end p-terminus. The physical map was integrated with the genetic map by the direct placing cDNA genetic markers that contained the RFLP marker sequence to the chromosomes [37]. Nevertheless, molecular cytogenetic studies on *Ae. aegypti* mitotic chromosomes remain challenging. The current physical map has relatively low resolution and includes ∼180 markers [11]. Only ∼31% of the *Ae. aegypti* draft genome assembly has been placed to chromosomes, but without order and orientation. In contrast, a physical map of the malaria vector *An. gambiae* includes more than 2000 markers and covers about 88% of the genome [10], [38].

Successful physical mapping for any organism relies on a robust source of high-quality, easily obtainable chromosome preparations. Recently we discovered that imaginal discs (IDs) of 4^th^ instar larva can be an excellent source for a high number of large, easily spreadable, banded chromosomes. In this study, we optimized all cytogenetic procedures required for the successful *in situ* hybridization. Idiograms for each individual chromosome at the metaphase stage have been developed. Based on the banding pattern, 10 BAC clones and a 18S rDNA probe were mapped to their precise chromosomal positions. We propose to use this new cytogenetic tool for the detailed physical mapping of the *Ae. aegypti* genome.

## Methods

### Mosquito strain

In this study, we used the Liverpool strain, a parental strain for the Liverpool IB-12 strain, which was used for sequencing the *Ae. aegypti* genome [11]. Eggs were hatched at 28°C, and after several days, 2^nd^ or 3^rd^ instar larvae were transferred to16°C to obtain a high number of mitotic divisions in IDs.

### Slide preparation

For *in situ* hybridization and idiogram development, slides were prepared from 4^th^ instar larvae of *Ae. aegypti.* Before dissection, larvae were placed on ice for several minutes, then transferred to a slide with a drop of cold hypotonic solution (0.5% sodium citrate), and after that dissected under a Olympus SZ microscope (Olympus America, Inc., Melville, NY, USA). Larvae were decapitated, and cuticle from the ventral side of the larval thorax was slightly cut by dissecting scissors (Fine Science Tools, Foster City, CA, USA). The cuticle was opened to expose the IDs to treatment in hypotonic solution for 10 min. Hypotonic solution was removed using filter paper, and larvae were treated with Carnoy's solution (ethanol/glacial acetic acid in 3∶1 ratio) for 1 min. After Carnoy's application, IDs immediately turned white and became easily visible under the microscope. Using dissecting needles (Fine Science Tolls, Foster City, CA, USA), IDs were isolated from larvae, transferred to another slide in a drop of 50% propionic acid, and covered with a 22x22-mm cover slip. After 10 min of propionic acid treatment, IDs were squashed and briefly analyzed using an Olympus CX41 microscope (Olympus America, Inc., Melville, NY, USA) at ×200 magnification. Slides suitable for *in situ* hybridization, which had >50 chromosome spreads, were then placed in liquid nitrogen, and cover slips were removed. Slides were dehydrated in a series of ethanol (70%, 80%, 100%) and air dried. The percentage of the slides suitable for *in situ* hybridization was >90%.

For the analysis of mitosis dynamics in IDs and brain ganglia, larvae were fixed in Carnoy's solution (ethanol/glacial acetic acid in 3∶1 ratio). After 24 hours, IDs and brain ganglia were dissected from larvae and squashed in 50% propionic acid. Small drops of lactic acid were placed on each corner of the cover slip to prevent slides from drying. Slides were analyzed under the Olympus CX41 microscope at x400 magnification.

### DNA probe and Cot1 fraction preparation

BAC clone DNA was isolated using the Qiagen Large Construct kit (Qiagen Science, Germantown, MD, USA). BAC-DNA was labeled by nick translation. Each reaction mix contained: 1 µg of DNA; 0.05 mM each of unlabeled dATP, dCTP, and dGTP, and 0.015 mM of dTTP (Fermentas, Inc., Glen Burnie, MD, USA); 1 µl of Cy3 or Cy5 dUTP (GE Healthcare UK Ltd, Buckinghamshire, UK); 0.05 mg/ml of BSA (Sigma, St. Louis, MO, USA); 5 µl of 10x nick translation buffer; 20 u of DNA polymerase I (Fermentas, Inc., Glen Burnie, MD, USA); and 0.0012 u of DNAse I (Fermentas, Inc., Glen Burnie, MD, USA). DNA polymerase/DNAse ratio was selected empirically to obtain the probe with the size range from 300 to 500 bp.

To obtain a C_0_t1 DNA fraction, the genomic DNA was isolated from adult *Ae. aegypti* mosquitoes using a blood and cell culture maxi kit (Qiagen Science, Germantown, MD, USA). DNA was digested by DNAse I with a concentration 0.0002 u/µl (Fermentas, Inc., Glen Burnie, MD, USA) to obtain fragments <100 bp. After that, DNA was denatured at 97°C for 10 min, and DNA fragments were allowed to reassociate in TE buffer for 1 hour at 37°C. Then single-stranded DNA was digested using S1 nuclease (Invitrogen Corporation, Carlsbad, CA, USA) with a concentration of 2.58 u/µl for 15 min at 37°C. Double-stranded C_0_t1 DNA fraction was collected by standard ethanol precipitation for further application.

### Fluorescent *in situ* hybridization

Fluorescent *in situ* hybridization (FISH) was performed using a standard protocol [39]. Slides were pretreated with 0.1 mg/ml of pepsin (USB corp., Cleveland, Ohio) for 5 min at 37°C; denatured in deionized 70% formamide in 2xSSC at 72°C for 5 min; and dehydrated in an alcohol series (70%, 80%, and 100%) for 5 min each. Hybridization mix contained 50% formamide, 10% dextran sulfate (Sigma, St. Louis, MO, USA), 200 ng of each probe per slide, and 4 µg of C_0_t1 DNA fraction. To eliminate nonspecific hybridization to the chromosomes, the probe was prehybridized with C_0_t1 fraction in a tube at 37°C DNA for 30 min. After that, the final 10 µl volume of hybridization mix per slide was overlaid with a 22x22 cover slip and glued by rubber cement. Hybridization on the slide was performed at 37°C in a dark humid chamber over night. Afterward, the slides were washed in a Coplin jar with 0.4x SSC, 0.3% Nonidet-P40 at 72°C for 2 min, and in 2x SSC, 0.1% Nonidet-P40 at RT for 5 min. Slides were thereafter counterstained using 1 µM YOYO-1 iodide solution (Invitrogen Corporation, Carlsbad, CA, USA) in 1× PBS for 15 min and enclosed under antifade Prolong Gold reagent (Invitrogen Corporation, Carlsbad, CA, USA) by a cover slip. Slides were analyzed using a Zeiss LSM 510 Laser Scanning Microscope (Carl Zeiss Microimaging, Inc., Thornwood, NY, USA) at ×1000 magnification.

### Image processing

To develop idiograms, the best images of the chromosomes stained with YOYO-1 were selected. The colored images were inverted in black and white images and contrasted in Adobe Photoshop as described before [40]. The chromosomal images were straightened using ImageJ program [41] and were aligned for comparison. In total, 150 chromosomes at various stages of condensation were analyzed.

### Measurements and statistics

The sizes of IDs were measured using an SZ dissecting microscope (Olympus America Inc., Melville, NY, USA). The lengths of the chromosomes were measured using Zen 2009 Light Edition software [42]. The statistic analysis was performed using the JPM8 software program at 95% confidence intervals Heiberger [43].

## Results

### Polytene chromosomes in *Aedes aegypti*

To obtain polytene chromosomes for cytogenetic analysis of *Ae. aegypti*, we have screened several tissues from different developmental stages including 4^th^ instar larvae, pupae, and adults. Polytene chromosomes were found in salivary glands, Malpighian tubules, and ovarian nurse cells. However, polytene chromosomes had poor banding patterns and formed multiple ectopic contacts in all examined tissues. To improve the quality of the polytene chromosomes, we maintained the larval stages at 16°C. Reduced rearing temperature was effectively used to improve the quality of the polytene chromosome in salivary glands of *Culex pipiens* [44]. In our study, we did not detect any such improvement in the polytenization level or chromosome structure in *Ae. aegypti*. Finally, we confirmed that polytene chromosomes in *Ae. aegypti* are not suitable for the physical mapping of the genome.

### Mitotic chromosomes in *Aedes aegypti*

**Figure 1:**
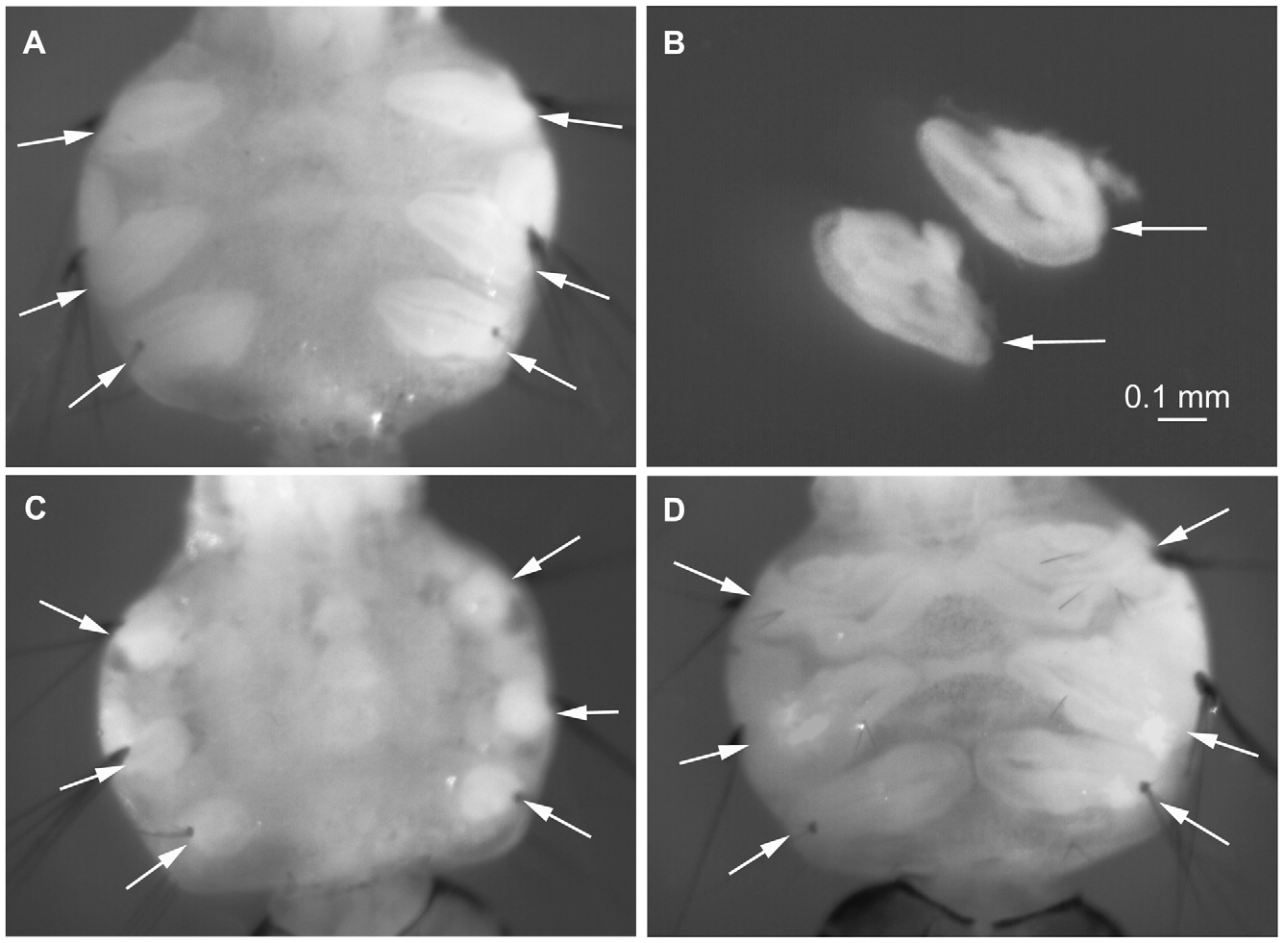
IDs at different stages of their development. The IDs of optimal size (A, B), underdeveloped (C) and overdeveloped IDs (D) are shown. The location of the IDs under the cuticle in thoracic segments of 4^th^ instar larvae (A, C, D) and dissected IDs (B) are indicated by arrows.

**Figure 2:**
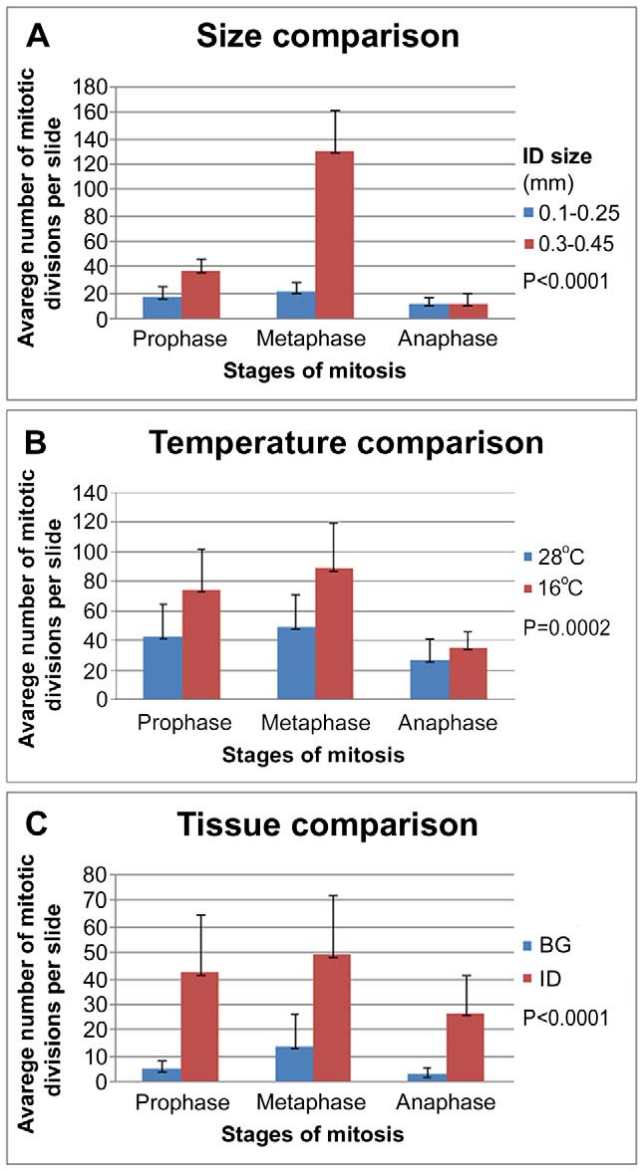
The dynamics of mitosis in IDs and brain ganglia. The mitosis in IDs of two different sizes (A), in IDs at two different temperatures (B) and in one ID and two brain ganglia (C) are compared.

In addition to polytene chromosomes, we analyzed mitotic chromosomes from IDs and brain ganglia. Six IDs, which will develop into legs at the adult stage, are located right under the cuticle on the ventral side of the thorax in larva (Fig. 1). Although IDs become visible under the dissecting microscope from the 2^nd^ instar larval stage, the best stage for the chromosome preparation is 4^th^ instar larvae when IDs start to develop into legs and accumulate large numbers of mitotic divisions. IDs at different stages of their development are shown in Fig. 1. The size of IDs in 4^th^ instar larvae ranged from 0.1 to 0.8 mm. Fig. 1D represents overdeveloped IDs, which are not suitable for slide preparation because of the abundance of already differentiated tissues. In this study, the number of mitotic divisions per slide was compared between: 1) IDs of two sizes--0.1*–*0.25 mm and 0.3-0.45 mm (Fig. 2A); 2) IDs from larvae reared at 28°C and 16°C (Fig. 2B); and 3) one ID and two brain ganglia (Fig. 2C). The largest number of mitotic divisions (∼175) was detected in IDs with an oval shape and length of 0.3*–*0.4 mm (Fig. 1A, B). The 16°C temperature stimulated the accumulation of ∼1.5 times higher number of mitotic divisions per slide as compared to the normal temperature (Fig. 2B). Finally, our comparison indicated a ∼6 fold difference in number of mitotic divisions between one ID and two brain ganglia (Fig. 2C). This parameter is extremely important for utilizing chromosome preparations for successful *in situ* hybridization.

**Figure 3:**
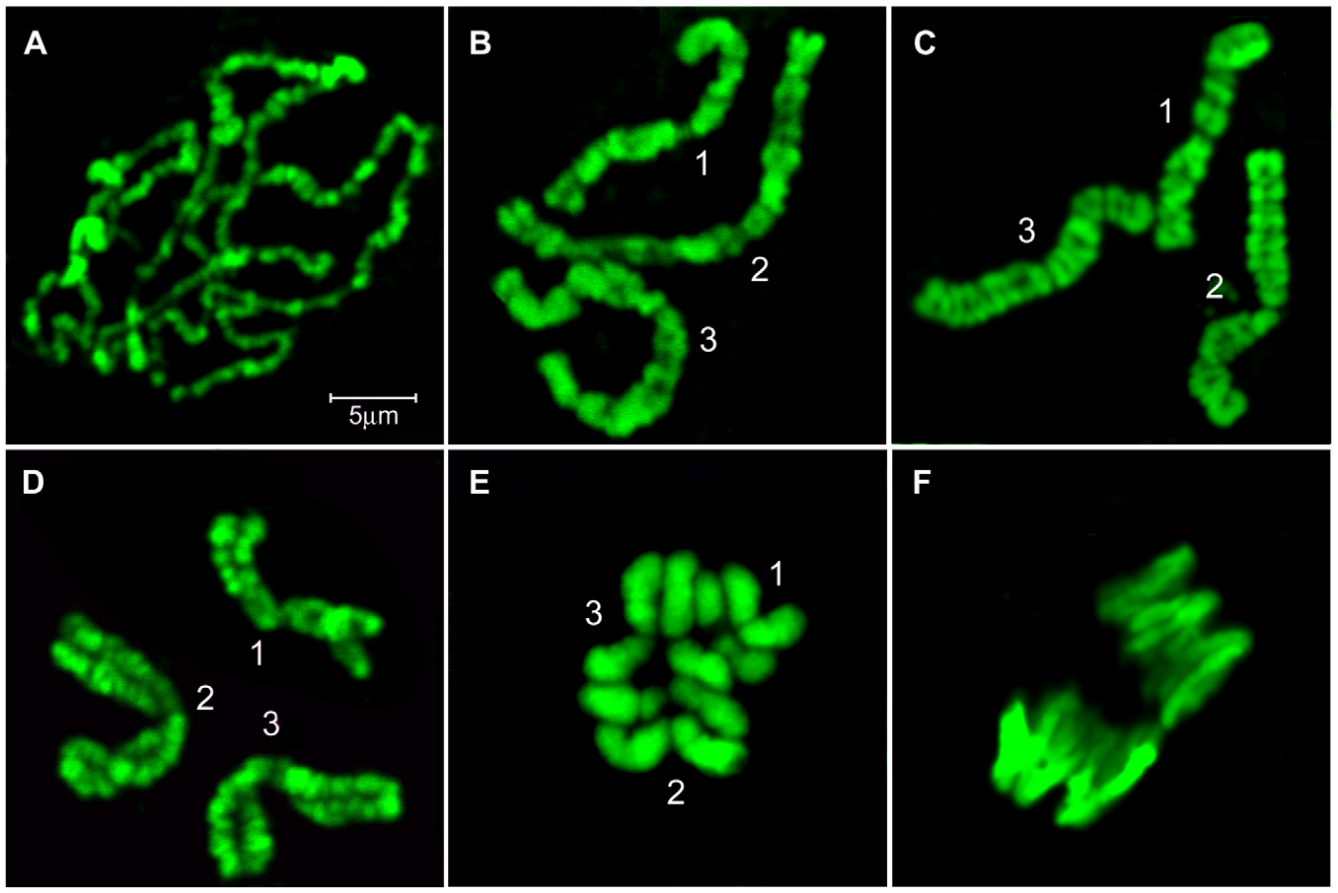
Chromosomes from IDs at different stages of mitosis. Prophase (A-C), prometaphase (D), metaphase (E), and anaphase (F) are shown. Chromosomes 1, 2, and 3 are indicated by numbers.

The major phases of mitosis in IDs of *Ae. aegypti* are shown in Fig. 1: prophase (A-C) prometaphase (D); metaphase (E) and anaphase (F). The interesting feature, which characterizes mitosis in *Ae. aegypti*, is that homologous chromosomes have strong somatic synapsis during interphase and stay paired up to early metaphase (Fig. 3A-D). As a result of chromosomal pairing, only three separate chromosomes can be detected in all cells at the early mitotic stages. At metaphase, homologous chromosomes finally segregate from each other, and the visible number of chromosomes becomes equal to 6 (Fig. 3E). The synapsis of the homologous chromosomes in *Aedes* cells has been described before [45]. Prometaphase and metaphase chromosomes (Fig. 3D, E) are the most abundant in IDs (∼42%) and easily identifiable by their relative lengths and morphological characteristics. Long prophase chromosomes (Fig. 3A-C), which are present in IDs at the level of ∼35%, are convenient for the mapping and orientation of relatively short scaffolds with sizes ∼1 Mb. Thus, ∼77% of all chromosome spreads on the preparations of squashed IDs can be utilized for the cytogenetic analysis and the physical mapping of *Ae. aegypti* genome.

### Idiograms of the imaginal disc chromosomes of *Aedes aegypti*

Another important feature of the mitotic chromosomes in IDs of *Ae. aegypti* is a clearly visible and reproducible banding pattern that can be used for developing idiograms--the diagrammatical representation of the chromosomes. In this study, idiograms for mid-metaphase chromosomes, the most convenient stage for chromosome recognition, have been developed. To calculate the correct proportion of the idiograms, chromosomes were measured using Zen2009 Light Edition software [42]. The results of these measurements are summarized and compared with previous data in Table 1. The average lengths of the chromosomes were 7.15 µm, 9.46 µm, and 8.36 µm for chromosomes 1, 2, and 3, respectively. The relative lengths of the chromosomes were 28.48%, 37.93%, and 33.39%. Centromeric indexes (the relative length of the p-arm) were 46.92%, 48.61%, and 47.42%, respectively, for chromosomes 1, 2, and 3. Therefore, all three chromosomes should be considered as metacentric regarding current chromosomal nomenclature [23]. The average lengths of the chromosomes from IDs at the metaphase stage were just slightly ∼0.8 µm bigger than that from ATC-10 line [16]. The relative lengths of the chromosomes were found to be very similar to the chromosomes from brain [18], spermatogonia [22], and ATC-10 line [16]. Interestingly, the centromeric indexes in our study were more similar to that from brain and spermatogonia than to the cell line (Table 1).

**Table 1:** Comparison of the *Aedes aegypti* chromosomes from different sources.

Source of chromosomes	IDs	BG	SG	ATC-10
**Reference**	Current	[18]	[22]	[16]
**Chromosome 1** Average length, µm	7.1	NA	NA	6.37
Relative length, %	28.5, P<0.0001	27.1	27.9	27.3
Centromeric index, %	46.9, P = 0.0039	NA	46.9	46.3
**Chromosome 2** Average length, µm	9.5	NA	NA	8.61
Relative length, %	37.9, P<0.0001	38.2	38.3	36.9
Centromeric index, %	48.6, P = 0.0552	NA	48.2	47.7
**Chromosome 3** Average length, µm	8.4	NA	NA	8.33
Relative length, %	33.5, P<0.0001	34.7	33.8	35.7
Centromeric index, %	47.4, P = 0.0025	NA	45.6	49.3

NA – not applicable; **ID** - imaginal discs; **BG** – brain ganglia; **SG** – spermatogonia;

**ATC-10** – cell line

**Figure 4:**
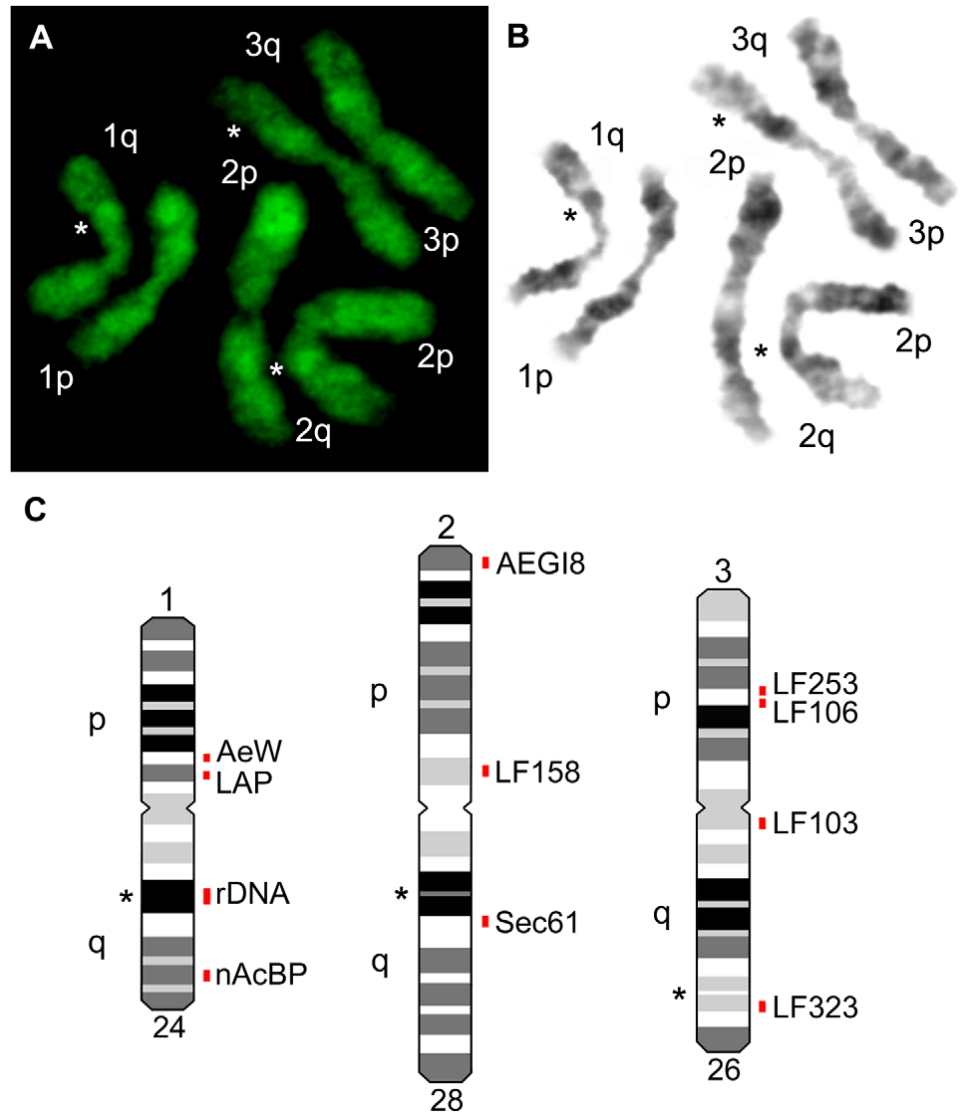
Development of idiograms for the ID chromosomes. Initial color images of YOYO-1 stained chromosomes (A), negatives of the same images converted to grey scale (B) and idiograms with BAC clone locations (C) are shown. Chromosomes 1, 2, and 3 are indicated by numbers, p – the short arm, q- the long arm of the chromosome. Chromosomal landmarks are shown by stars. The positions of the BAC clones contained specific genetic markers (AeW, AEGI8, LF103, etc.), and 18S rDNA are indicated by red bars.

**Figure 5:**
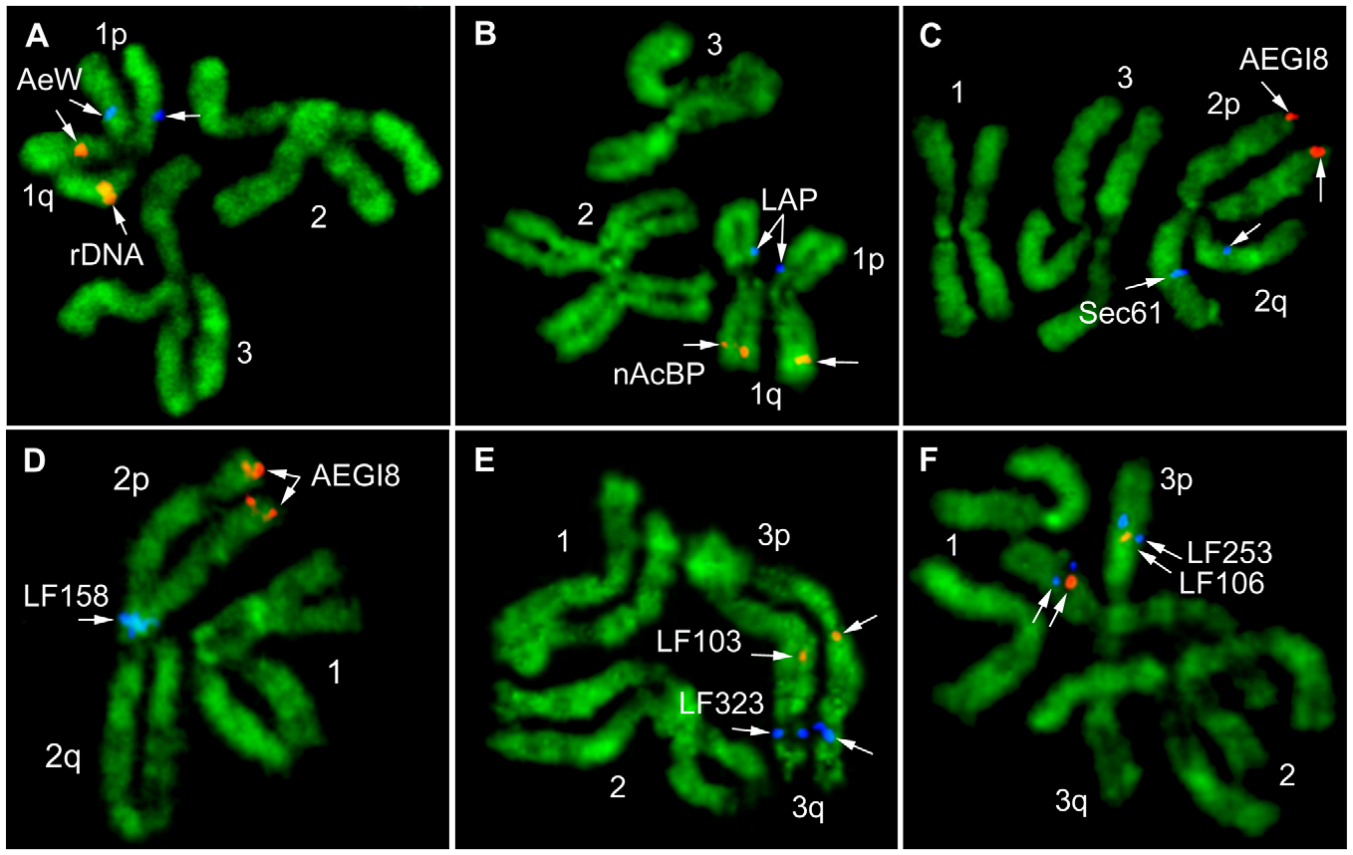
Examples of *in situ* hybridization of DNA probes to ID chromosomes. 18S rDNA probe and 10 BAC clones were mapped to the chromosomes 1 (A, B); 2 (C, D); and 3 (E, F). Chromosomes are indicated by numbers, p – the short arm, q- the long arm of the chromosome. The positions of the signals on the chromosomes are indicated by arrows. The BAC clones are named by genetic markers which they are carrying (Table 2).

Fig. 4 shows the major steps of the idiogram development. The images of the YOYO-1 stained chromosomes (Fig. 4A) were converted in black and white images (Fig. 4B) and further contrasted in Adobe Photoshop [40] to obtain clear banding patterns. After that, chromosomes were straightened using Image J program plug-in [41] and aligned to each other for the pattern comparison. In total, 150 chromosomes were analyzed. Chromosomal arms were first determined by FISH of the BAC clones with known chromosomal positions (Fig. 5). These BAC clones contained genetic markers previously genetically mapped to the chromosomes [46]. Based on the human cytogenetic nomenclature, we determined bands with 4 different intensities – intense, medium intensity, low intensity, and negative [47]. The total number of bands per three chromosomes at mid metaphase was equal to 78. The following regions can be used as cytogenetic landmarks for the chromosomal arm recognition: intense band in the middle of the 1q arm, intense double band in the 2q arm, and 2 low intense bands in the area next to the telomeric band on the 3q arm (Fig. 4). These regions have consistent distinct morphology and can be easily utilized for the chromosomal arm recognition.

**Table 2.List:** of the probes hybridized to the chromosomes from imaginal discs of *Aedes aegypti.*

Probe	Genetic marker	Accession #	Genetic location, cM	Scaffold	Chromosome
NDL.58C3	AeW	U73826	1-29.7	1.71	1p
NDL.18P1	LAP	M95187	1-36.6	1.192	1p
18S rDNA	NA	AY988440	NA	1.6997	1q
NDL.109E9	nAcBP	AY040341	1-44.5	1.1051	1q
NDL.106A1	AEGI8	AF326340	2-0.0	1.145	2p
NDL.40I24	LF158	BM005485	2-36.7	1.1168	2p
NDL.52E23	Sec61	AF326338	2-37.8	1.122	2q
NDL.30K18	LF253	T58331	3-16.7	1.146	3p
NDL.67B23	LF106	BM005490	3-26.1	1.488	3p
NDL.5F19	LF103	BM005488	3-23.5	1.766	3q
NDL.19M6	LF323	BM005507	3-43.7	1.86	3q

NA - not applicable.

### Physical mapping on chromosomes from imaginal discs of *Aedes aegypti*

To test the reliability of chromosomal banding patterns for physical mapping, 10 BAC clones (Table 2) were placed to their precise chromosomal positions on idiograms (Fig. 4C) by FISH. All BAC clones contained genetic markers (Jimenez et al., 2004), and their positions on the chromosomes were predicted by previous genetic mapping [29]. In our study, most of the BAC clones followed the order of the previous genetic mapping. Only one BAC clone with genetic marker LF103 was found in slightly different order. The expected position of this BAC clone was between genetic markers LF253 and LF106 on the 3p arm. The actual position of this BAC clone was close to the centromere on 3q arm. Thus, the idiograms for the mitotic chromosomes from the ID cells of *Ae. aegypti*, which are presented here, can be successfully utilized for the physical mapping of the *Ae. aegypti* genome.

## Discussion

The genome of *Ae. aegypti* has several features that make physical mapping and genome assembly difficult. First, *Ae. aegypti* and other aediines have the largest genomes within the Culicidae family investigated thus far [11]. Second, the *Ae. aegypti* genome is extremely enriched with DNA repeats: about half of the genome consists of transposable elements. Third, *Ae. aegypti* lacks well-developed spreadable polytene chromosomes [14], [15]. Initial physical mapping of the *Ae. aegypti* genome was performed on metaphase chromosomes from the ATC-10 cell line [16]. Using FLpter, a fractional length from the p-terminus (short arm telomeric end) for measuring the location of the signal on each chromosome, provided a very approximate localization on the chromosomes. In addition, using chromosomes from the permanent (immortalized) cell lines for the genome mapping can be misleading because these cells usually accumulate chromosomal rearrangements. Two large chromosomal translocations were described in the ATC-10 line [16]. It has been shown that in the cell culture of *Ae. albopictus* ∼30% of the cells were tetraploid and 30% of the diploid cells had chromosomal aberrations [48]. As a result of these difficulties and limitations, less than one third of the *Ae. aegypti* draft genome assembly has been placed to chromosomes mostly based on results from genetic recombination mapping efforts, but without order and orientation [11].

Using chromosomes from IDs of 4^th^ instar larvae for the physical mapping of the *Ae. aegypti* genome as proposed here will help to overcome the above problems. Preparation of the chromosome spreads from IDs is a simple, robust procedure. In this study more than 90% of the slides were suitable for *in situ* hybridization. The number of the chromosome spreads per slide in IDs was also high. We were able to find ∼150 chromosome spreads per individual ID at the stages appropriate for the mapping. Finally, presence of these chromosomes in the IDs makes any individual mosquito at the larval stage available for cytogenetic analysis and allows avoiding having to use cell culture chromosomes for the physical mapping.

The chromosome spreads from ID cells have two features important for physical mapping. First, chromosomes at all stages of mitosis have reproducible banding pattern which can be easily visualized by fluorescent staining with YOYO-1. Band-based physical mapping can be easily applied to these chromosomes instead of previously used distance–based mapping (FL-pter, fractional length from the p-terminus) [16]. This approach will lead to the precise positioning of the BAC clones and genome assemblies on the chromosomes. In addition to band-based mapping, the direct labeling of the DNA probe, which we used in our study, provides more precise location of the signal on the chromosome as compared to antibody-detected probes used before [16]. Second, the significant number of chromosome spreads in IDs (up to ∼30%) might be found at early stages of mitosis. Prometaphase and especially prophase chromosomes reflect significantly lower chromatin condensation and can be utilized for the orientation of relatively short scaffolds up to size ∼1 Mb. The average size of the scaffolds in the current *Ae. aegypti* genome assembly is 1.5 Mb [11]. In order to map and orient scaffolds on the chromosomes, the probes for the BAC clones from the opposite sides of the scaffolds must be labeled with two different colors. This approach was successfully used for the mapping of *An. gambiae* heterochromatic scaffolds [38].

Recently maps for mitotic chromosomes were created and successfully used for the physical mapping of *Dr. melanogaster* heterochromatin [49], [50], [51], [52], [53]. Among other organisms, the most detailed cytogenetic analysis was performed for human and mammalian genomes [47]. The highly populated FISH-based physical maps of mammalian genomes included 9528 and 851 markers for human and canine, respectively [54], [55]. The importance of chromosome-based physical mapping for comparative genomics was recently emphasized by H. Lewin and coauthors in the article titled “Every genome sequence needs a good map” [56]. The authors suggested looking “back in the future” for developing high-resolution physical maps as an important framework for genome annotation and evolutionary analysis. Finding an appropriate source of chromosomes and developing chromosomal idiograms, as conducted in this study, is the first important step toward the assembly and further utilization of the genomic information for the yellow fever mosquito *Ae. aegypti*.
